# Benchmarking classification abilities of novel optical photothermal IR spectroscopy at the single-cell level with bulk FTIR measurements†

**DOI:** 10.1039/d4ay00810c

**Published:** 2024-07-08

**Authors:** Paul I. C. Richardson, Malcolm J. Horsburgh, Royston Goodacre

**Affiliations:** a Centre for Metabolomics Research, Department of Biochemistry, Cell and Systems Biology, Institute of Systems, Molecular and Integrative Biology, University of Liverpool BioSciences Building, Crown St Liverpool UK roy.goodacre@liverpool.ac.uk; b Microbiology Research Group, Institute of Systems, Molecular and Integrative Biology, University of Liverpool BioSciences Building, Crown St Liverpool UK

## Abstract

Fourier-transform infrared (FTIR) spectroscopy is a simple, fast and inexpensive method with a history of use for bacterial analysis. However, due to the limitations placed on spatial resolution inherent to infrared wavelengths, analysis has generally been performed on bulk samples, leading to biological variance among individual cells to be buried in averaged spectra. This also increases the bacterial load necessary for analysis, which can be problematic in clinical settings where limiting incubation time is valuable. Optical photothermal-induced resonance (O-PTIR) spectroscopy is a novel method aiming to bypass this limitation using a secondary lower wavelength laser, allowing for infrared measurements of a single bacterium. Here, using *Staphylococcus capitis*, *Staphylococcus epidermidis* and *Micrococcus luteus* strains as a model and FTIR as a benchmark, we examined O-PTIR's ability to discriminate single-cell samples at the intergenetic, interspecific and intraspecific levels. When combined with chemometric analysis, we showed that O-PTIR is capable of discriminating different between genera, species and strains within species to a degree comparable with FTIR. Furthermore, small variations in the amide bands associated with differences in the protein structure can still be seen in spite of smaller sample sizes. This demonstrates the potential of O-PTIR for single-cell bacterial analysis and classification.

## Abstract 2

Optical photothermal IR spectroscopy (O-PTIR) presents new opportunities for single-bacterium analysis. Here, we assess its ability to classify bacteria at various levels of similarity.

## Graphical abstract

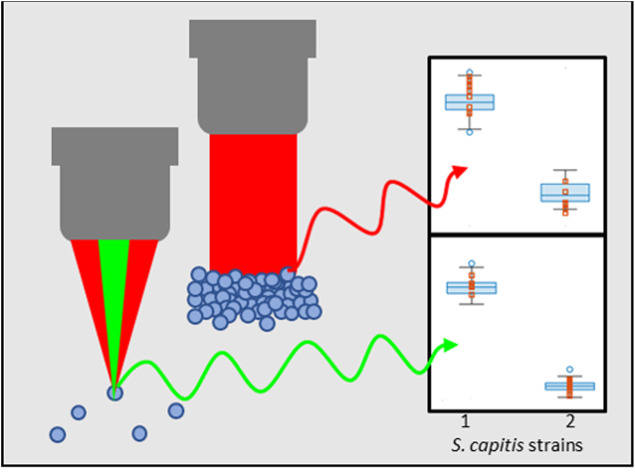

## Introduction

Fourier-transform infrared (FTIR) spectroscopy is a common analytical technique that has been employed in bacterial analysis for several decades.^1^ As a non-destructive and relatively simple method, it is able to provide a spectrum containing a holistic phenotypic measure of the sample being analysed^2^ through the probing of chemical groups present and their relative concentrations. Furthermore, it requires very little sample preparation past the bacterial growth stage. Since intact cells are measured, the only required step after separating cells from the growth medium is normalising the bacterial concentration and ensuring that the sample is dry, due to the interference from water. This also makes FTIR extremely inexpensive to operate, as no particular solvents or extreme conditions (*e.g.* low temperature for NMR spectroscopy) are necessary, and the sample plates used for analysis are reusable. However, FTIR is limited by its diffraction limit engendered by infrared light. Indeed, mid-IR wavelengths range between 2.5 and 25 μm (400–4000 cm^−1^), placing the smallest spatial resolution possible using conventional methods firmly in the micron range and thus rendering single bacterium analysis significantly more difficult.^3^ As such, all FTIR analyses are generally performed on bulk samples. While bulk sampling provides important information about bacteria, it limits analysis to bacteria that can be cultured while burying potentially important biological variance in a single average spectrum.

Improved spatial resolution was originally obtained using FTIR microscopy which, as its name implies, combines FTIR spectroscopy and microscopy to image smaller cells.^4^ The infrared light is focused using a series of Cassegrain mirrors, and the resolution can be improved through more sensitive photodetectors, the use of germanium ATR crystals, which have an increased refractive index, and more powerful synchrotron radiation or newly developed quantum cascade lasers (QCLs).^5^ Oversampling can also be employed to improve resolution artificially. Using these tools, the spatial resolution of FTIR can be improved to ∼10 μm, allowing for the analysis of single eukaryotic cells^6–8^ and small groups of bacteria.^9–11^ This, however, is still limited by the inherent diffraction limit of IR light and the challenges each of these modifications entail. Synchrotron radiation often requires access to dedicated facilities, and the more sensitive photodetectors require extra cooling either electrically or with liquid nitrogen. Longer exposure times can also be used to improve the signal-to-noise ratio; however, this increases experiment time and risks causing radiation damage to the sample.

One of the more recent methods used to bypass this limitation is optical photothermal IR (O-PTIR) which utilises the mirage effect,^12,13^ an effect by which a fast, temporary change in the refractive index of a sample is induced by thermal expansion from excitation. Here, this effect is induced by monochromatic infrared radiation and the change in refractive index results from the excitation of vibrational modes of molecular bonds by this radiation.^14^ As such, a mid-IR spectrum can be indirectly obtained by probing the intensity of the mirage effect at various wavelengths. This effect and its applications have been studied by various groups using homemade systems.^15,16^ The mIRage system is a recent commercial system combining a series of QCLs as an excitation source with a 532 nm laser to probe the mirage effect, and has allowed for significantly higher access to this technology.^17–20^ As the diffraction limit is now dependent on the probe rather than the IR excitation wavelength, the resolution improved to ∼0.5 μm, allowing for single bacterium measurements.

The main trade-off that must be considered when comparing O-PTIR to FTIR spectroscopy is the significantly decreased sample concentration. Rather than the globar light used in conventional FTIR, the infrared source in O-PTIR is a quantum cascade laser. This, alongside the probe laser, already drastically increases the energy being irradiated onto the sample. Additionally, the smaller sample size requires that a similar amount of energy be directed into a smaller radius. These factors drastically increase the risk of sample degradation, and in turn force a careful balance between ensuring sufficient laser power to obtain an acceptable signal and avoiding burning the sample in the process.

Here, we aimed to compare FTIR and the new mIRage O-PTIR instrument for their ability to discriminate between a group of Gram-positive bacterial strains. Five strains of bacteria were used: two strains of *Staphylococcus capitis*, two strains of *Staphylococcus epidermidis*, and one strain of *Micrococcus luteus*. These samples allow for three levels of distinction: the intergenetic level (*Micrococcus vs. Staphylococcus*) remaining nonetheless similar in shape, interspecific level (*S. capitis vs. S. epidermidis*) and intraspecific level (strain specific differentiation). Furthermore, we examined the sources of separation within each comparison by FTIR and O-PTIR with the aim of ascertaining whether individual sample variance affects discrimination, alongside whether the use of single cell analysis leads to differences not seen by bulk measurement and *vice versa*.

## Methodology

### Growth and sample preparation

*S. epidermidis* strains 11047 & RP62A, *S. capitis* subspecies *capitis* (DSM20327) and subspecies *urealyticus* (ATCC 49325), and *Micrococcus luteus* (NCTC 2665) were all obtained from the Horsburgh group. From glycerol stocks, these were grown on LB agar (Formedium, King's Lynn, Norfolk, UK) plates at least twice prior to experimental use. Bacteria were then grown in five biological replicates in LB broth (Formedium). In all cases, *Staphylococcus* samples were incubated at 37 °C overnight (∼18–20 h) and *Micrococcus* samples were incubated for two nights (∼42–44 h) to ensure that both reached the stationary phase of growth.

To prepare bacteria for analysis, aliquots (2 mL) of the broth were centrifuged at 5000*g* for 5 min, and the broth was removed. The bacterial pellets were then re-suspended in 1 mL deionised H_2_O to wash away any remaining broth following a protocol created by Lima *et al.*,^17,19^ and centrifuged again to allow the water to be removed once again. Bacteria were then re-suspended in water and diluted such that the optical density of each sample at 600 nm was 15.

### Data collection

For FTIR, three 20 μL aliquots of each sample were spotted on a Si 96-well plate in a randomised order and left to dry overnight at room temperature. The plate was analysed using a Bruker Invenio FTIR spectrophotometer in absorbance mode equipped with an HTS-XT high throughput plate reader spectrometer (Bruker, Coventry, UK) with a spectral range of 4000–400 cm^−1^ and a wavelength independent spot size of 3 mm. Each spot was analysed in three replicates, each time using different portions of the spot that did not overlap, and each spectrum was composed of 64 acquisitions (co-adds) and 2512 bins.

For the O-PTIR experiment, each sample was diluted by a factor of ∼100 and a 3 μL aliquot of each sample was spotted on a CaF_2_ slide. Two biological replicates for each *Staphylococcus* spp. were analysed twice over two days on a mIRage optical PTIR spectrometer (PhotoThermal Spectroscopy Corp, Santa Barbara, CA, USA), using an IR quantum cascade laser with a range of 1797.5–803.5 cm^−1^ for excitation and a green laser (532 nm) to probe the resulting thermal expansion, alongside one *M. luteus* control replicate. Staphyloccoci and micrococci are known to aggregate into bunches, making true single cells difficult to locate. Additionally, as these single cells were generally found to be dead or lysed (presumably either due to the use of deionised H_2_O in the washing steps causing osmotic shock or the expected presence of dead cells in a culture reaching the stationary phase) and subsequently ejected from their cluster, the definition of “single cells” was expanded to include discrete groups of 2–5 cells. The mIRage's auto-focus function was used to optimise the depth prior to each spectrum acquisition, and five 8 second acquisitions were averaged for each spectrum (498 total bins).

### Data analysis

The acquired FTIR spectra were first baseline corrected in OPUS software (provided by the manufacturer to control the spectrometer) using a rubber band algorithm, after which all data collected were transferred to Matlab R2020a (Mathworks, Natick, MA, USA) for data processing and analysis. FTIR spectra were scaled using an EMSC^21^ algorithm, after which the CO_2_ signals (2401–2275 cm^−1^ and 681–661 cm^−1^) were replaced with linear segments. Analysis was performed using mean centred principal component analysis (PCA), an unsupervised multivariate method aiming to reduce dimensionality by grouping variables (in this case, intensities at each wavenumber) into orthogonal PCs.^22^ These data were further manipulated using discriminant functional analysis (DFA), a supervised method which modifies PCA results obtained into a new orthogonal discriminant function, with the aim of maximising the separation between different classes.^23^ Here, classes were set as bacterial strains. To minimise the risk of overtraining the model, the number of PCs used was such that each contributed >1% to the total explained variance (TEV), and only the first four biological replicates were used to create the DFA model (48 spectra per class), with the fifth replicate acting as a test set (12 spectra per class).

O-PTIR spectra were first baseline corrected using an asymmetric least squares baseline correction algorithm,^24^ and EMSC scaled and smoothed using a Gaussian smoothing algorithm^25^ with a window width of 9. After this, spectra were once again analysed using mean centred PCA followed by DFA, once more ensuring that each PC contributed >1% to the TEV, and the last three spectra per day for each biological replicate were used as a test set.

## Results and discussion

### FTIR measurements

Although this experiment involves the comparison of FTIR and O-PTIR analysis, the aim was not to ascertain which one is superior, but to determine whether O-PTIR on single bacterial cells provide representative infrared spectra. Of course, the direct analysis of infrared absorption and the higher sample quantity provides FTIR with an advantage; O-PTIR is not expected to obtain superior results. However, the advantages conferred to FTIR allow us to use its result as an acceptable benchmark. In essence, any failure of discrimination by FTIR can also be expected in O-PTIR measurements and any limits found in O-PTIR can be placed in a more useful context.

**Fig. 1:**
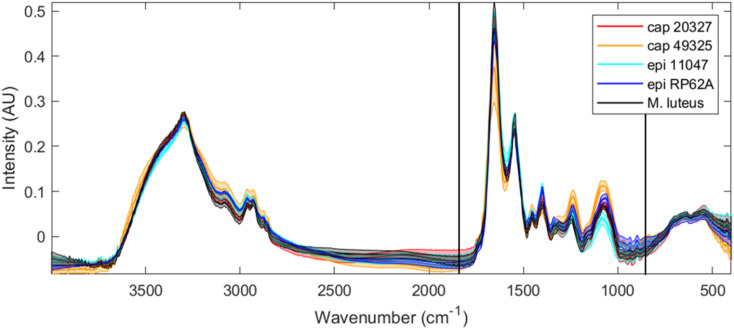
Full range FTIR spectra for all five strains tested (red: *S. capitis* 20327, orange: *S. capitis* 49325, light blue: *S. epidermidis* 11047, dark blue: *S. epidermidis* RP62A, and black: *M. luteus*) after rubber-band baseline correction, EMSC scaling and CO_2_ signal removal. The thicker line of each colour depicts the average (mean) spectrum for each strain, while the shaded area depicts the range of one standard deviation. The area between the two black vertical lines indicates the truncation performed for the data analysis, and this is the same spectral wavenumber range collected by O-PTIR.

Fig. 1 depicts the averaged FTIR spectra for each of the five strains examined, with a shaded area around each spectrum representing a range of one standard deviation at each point. Unsurprisingly, the spectra are, at first glance, quite similar. Bacteria can be simplified to mixtures of sugars, lipids, DNA and RNA, proteins and miscellaneous small molecules, with significant differences in genotype or activity being measured as slight differences in peak ratios or peak positions. The clearest spectral differences between bacterial classes can be found in *S. capitis* 49325, which has a larger range at the amide I peak (1650 cm^−1^), alongside increased intensities at the 1228 cm^−1^ peak ∼1050 cm^−1^ range. However, while these spectral differences can allow us to perhaps separate *S. capitis* 49325 from the rest, it is far more efficient to use chemometric methods to classify these strains and understand the phenotypical differences that exist between them.

**Fig. 2:**
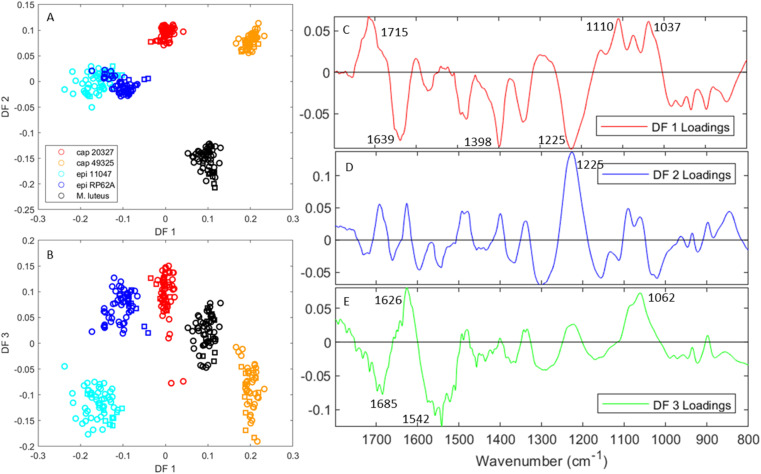
PC-DFA scores and loadings from a model containing the 1800–800 cm^−1^ range of the FTIR spectra for all five tested strains (red: *S. capitis* 20327, orange: *S. capitis* 49325, light blue: *S. epidermidis* 11047, dark blue: *S. epidermidis* RP62A, and black: *M. luteus*). The original PCA model contained 7 PCs and these accounted for 97.8% of the total explained variance (TEV). (A and B) The scores for DF 1 *vs.* 2 and DF 1 *vs.* 3 respectively, where circles of each colour represent the training set and squares, the test set. (C–E) The loadings for DF 1, 2 and 3 respectively.

Fig. 2 shows the chemometric separation obtained through principal component – discriminant function analysis (PC-DFA). PC-DFA is a supervised chemometric method built upon PCA, which mathematically alters the principal components (in this analysis the first 8 PCs) to maximise separation between classes and minimise separation within them, thus often drastically increasing discrimination ability. However, due to PC-DFA being a supervised method, there is a risk of creating an over-fitted model wherein false separation is obtained through noise and additional data cannot be fitted. To avoid this, the fifth biological replicate of each strain was excluded from the data set prior to model creation, and subsequently added as a test set to ensure that the model was not over-fitted; that is to say, if the model were to be generalised, the test set samples would co-locate with the training set samples from the same bacterial class.

Fig. 2A and B present the scores over DF 1 *vs.* 2 and DF 1 *vs.* 3 respectively using FTIR spectra truncated to match the 1800–800 cm^−1^ range of our O-PTIR instrument. While Fig. 2A demonstrates that DF 2 separates *M. luteus* (black) from the other strains, Fig. 2B shows full separation between all strains using DF 1 and 3. Moreover, what is also evident from these ordination plots is that the test set data (represented by squares in these plots) do indeed cluster with their respective groups and thus the clustering observed is real and not due to the model being overfit. Modelling using the entire 4000–400 cm^−1^ range can also be found in the ESI (Fig. S1†).

We can obtain further information about these scores by studying the loadings of each discriminant function. Fig. 2C–E present the loadings for DF 1, 2 and 3 respectively. Over DF 1, we see five main areas of interest: a positive peak at 1715 cm^−1^, three negative peaks at 1639, 1398 and 1225 cm^−1^, and a positive area containing two main peaks at 1110 and 1037 cm^−1^. The peak at 1715 cm^−1^ is typically associated with lipid C=O vibrations, which would indicate variance in the lipid bilayer. The peak at 1639 cm^−1^ is offset from amide I and likely represents a difference in protein secondary structures, notably a higher proportion of β-sheets in the *S. epidermidis* strains.^26,27^ The peaks at 1398 and 1225 cm^−1^, while strong, are challenging to attribute as the former is generally associated with C–H bending vibrations present in all organic molecules and the latter does not match any peak, instead being positioned on the side of amide III.^28^ This could further represent variance in the protein content of these strains or a phosphate vibration associated with DNA. Finally, the positive 1030–1110 cm^−1^ area is typically known as the carbohydrate region as C–O–C vibrations associated with sugars can be found there. However, it is also an area containing peaks associated with peptidoglycans originating from the cell wall^29^ and teichoic acids.^30^

DF 2, which mainly separates *M. luteus* from the *Staphylococcus* strains, is mostly dominated by a large positive peak at 1225 cm^−1^. Interestingly, the other protein associated peaks do not appear particularly strongly, implying that this signal is more likely to represent variance associated with DNA. DF 3, in turn, is dominated by peaks in the amide region (1685, 1626 and 1542 cm^−1^) and a peak in the sugar region at 1062 cm^−1^. This provides evidence that the main differences between the two *S. epidermidis* strains lie in the protein structures and carbohydrates.

### O-PTIR measurements

Having established that there were clear phenotypic differences between these bacteria using FTIR spectroscopy, and that both species and strain differentiation was possible, the next stage was to see if this translated to O-PTIR measurements on smaller numbers of bacterial cells.

**Fig. 3:**
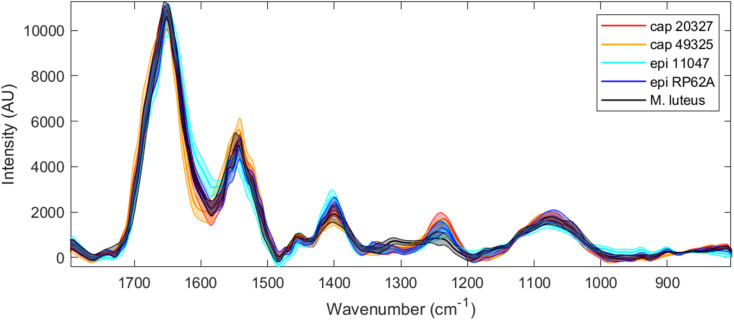
Full range O-PTIR spectra for all five strains tested (red: *S. capitis* 20327, orange: *S. capitis* 49325, light blue: *S. epidermidis* 11047, dark blue: *S. epidermidis* RP62A, and black: *M. luteus*) after asymmetric least-squares baseline correction, EMSC scaling and Gaussian smoothing. The thicker line of each colour depicts the average (mean) spectrum for each strain, while the shaded area depicts the range of one standard deviation.

Fig. 3 shows the averaged O-PTIR spectra for each strain examined alongside a shaded range of one standard deviation, similar to Fig. 1. Due to limitations of quantum cascade laser technology and prohibitive upfront cost to enable full-range scanning, only the 1800–800 cm^−1^ range was probed. Additionally, spectra were significantly less intense and not surprisingly noisier than in the FTIR dataset, leading to a need for smoothing and potential information loss as a result – this is especially visible in the 1050–1100 cm^−1^ region, where the two previously found clear peaks in FTIR have partly merged into one peak and an occasionally visible shoulder. This is overall perhaps expected as these spectra are collected from 1–5 cells, each containing a mere ∼1 pg of material, whereas FTIR spectra incorporate several million cells into each spectrum.

**Fig. 4:**
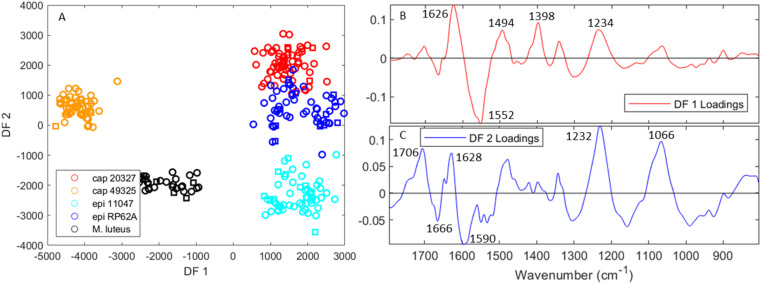
PC-DFA scores and loadings from a model containing O-PTIR spectra for all five tested strains (red: *S. capitis* 20327, orange: *S. capitis* 49325, light blue: *S. epidermidis* 11047, dark blue: *S. epidermidis* RP62A, and black: *M. luteus*). The original PCA model contained 7 PCs and explained 96.9% TEV. (A) The scores for DF 1 *vs.* 2, where circles of each colour represent the training set and squares, the test set. (B and C) The loadings for DF 1 and 2 respectively.

Fig. 4 depicts the PC-DFA scores plot and subsequent loadings for the separation of all five strains. Once more, spectra were divided into a training set used to create the model, and a test set (indicated by square symbols) added *a posteriori* to avoid overtraining. The test set was composed of the final three spectra per day, per biological replicate. This was possible as individual single cells or small groups were assumed to be separate samples from other cells, and limited the risk of bias relating from day-to-day or biological replicate variance.

Although the separation pattern is similar to that found in Fig. 2 especially along DF 1, there remain some interesting differences. Rather than separating the *S. capitis* and *S. epidermidis* strains from each other, DF 1 mainly separates *S. capitis* 49325 (and to a lesser extent *M. luteus*) from the other staphylococci, to the point where *S. capitis* 20327 and *S. epidermidis* RP62A overlap slightly. It should be noted when comparing the DFA plots from O-PTIR and FTIR that the scales of these ordination axes are a function of the *y*-axes of the spectra, which can be adjusted by normalisation of the spectra. Additionally, a separation of *S. epidermidis* RP62A into two distinct groups can be seen and these correspond to the two biological replicates that were tested. Interestingly, the other strains showed no significant separation based on biological replicates, which suggests that the spectral variance by growth is less than the variance within individual bacteria.

The DFA loadings outlining the sources of separation using O-PTIR (Fig. 4B and C) also contain encouraging similarities to the FTIR DFA loadings (Fig. 2C–E). DF 1 is dominated by two peaks at 1626 and 1552 cm^−1^, which are near matches to the two peaks in DF 3 for FTIR spectra (Fig. 2E) separating the two *S. capitis* species. It also contains input from the peaks at 1398 and 1225 cm^−1^, which provided separation of *M. luteus* from the other strains. Similarly, DF 2 (Fig. 4C), which separates the two *S. epidermidis* strains, contains several peaks in the amide and sugar regions which match the peaks of interest in the FTIR DFA loadings. Since DF 3 and 4 (Fig. S3A and B†) did not provide any useful separation, it seems that the three discriminant functions in the FTIR model were condensed into two for O-PTIR.

**Fig. 5:**
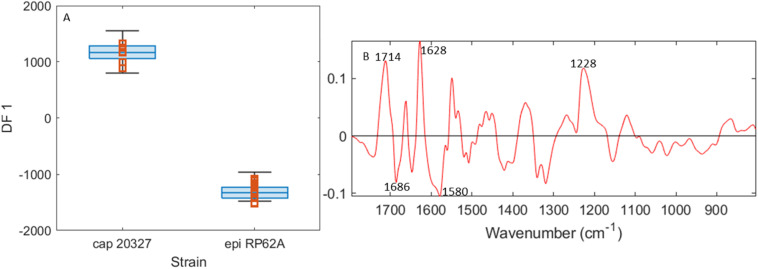
Results of PC-DFA modelling using O-PTIR spectra to compare *S. capitis* 20327 and *S. epidermidis* RP62A. For scores (A), the box and whisker plots depict the interquartile range (edges of the box), and the median (middle line in the box) and the extremes of the range are not counted as outliers (whiskers) for the training sets for each strain, while orange squares represent the test set. (B) The DF 1 loadings. The original PCA contains 8 PCs and explains 97.3% TEV.

To ascertain O-PTIR's ability to separate *S. capitis* 20327 and *S. epidermidis* RP62A, these two strains were modelled in a 1 *vs.* 1 comparison using DFA and are presented in Fig. 5 and S2.† It is clear that, where the separation is focused around these two strains, there is no challenge in separating these two strains. Furthermore, the loadings once more find spectral differences in the amide region, indicating differences in the protein structure, the peak at 1228 cm^−1^ associated with phosphates in DNA, and the peak at 1714 cm^−1^ previously associated with lipid C=O vibrations. On this basis, we conclude that the previous overlap between these two strains is a quirk of the model rather than a weakness of the instrument.

As described earlier, the novelty of the O-PTIR approach and the inherent limitations of signal strength relating from sample size and laser power restrictions highlighted the importance of understanding the quantity and quality of information that could be obtained from individual bacteria. The instrument has shown itself to be fully capable of delivering in that regard.^17–19^ When compared to FTIR, its closest analogue, and a now common spectroscopic technique for bacterial classification, as exemplified by using Bruker's IR Biotyper^31^ which uses FTIR to automatically identify bacterial strains, O-PTIR has not only shown similar discrimination ability at the species and even strain levels, but the similarities between the loadings of PC-DFA models for FTIR and O-PTIR data provided greater confidence in the biological relevance of the discrimination. Notably, the small differences in the amide I peak shape, associated with differences in the protein structure, were corroborated across both datasets. Additionally, although some of the minutiae of the 1100–1050 cm^−1^ area were not visible in the O-PTIR data, the peak shape was still sufficiently detailed to match the correlations found between the 1093 cm^−1^ and 1066 cm^−1^ peaks in the FTIR data.

There is great potential for this technology to be used in clinical settings as the access to single cell measurements could allow for drastically reduced incubation times or even allow culture-free analysis. Although the above results are promising, they remain limited as they artificially only consider bacteria at the stationary phase; bacterial FTIR spectra are known to undergo significant changes with transitions between lag, log, stationary and death phases,^32^ and there is no guarantee that bacteria analysed directly from a biopsy would be in a predictable stage of growth. To this end, the analysis of single cells at various stages would be a beneficial topic of future study, as it would add an extra dimension to any potential classification model. Once these are addressed then there will be significant potential for culture free analysis which is needed for clinical microbiological scenarios.

## Conclusion

FTIR spectroscopy is a simple and powerful method for analysing and classifying bacteria with decades of evidence in this regard.^33^ However, physical limitations have left it unable to probe bacteria at the single-cell level, which in turn has restricted its analysis to the average of a bulk sample at the cost of missing potentially important individual bacterial variance, as has been very recently exemplified by Lima and colleagues^19^ who showed using O-PTIR spectroscopy that it is now possible to probe individual cells of *Bacillus* spp. and observe phenotype heterogeneity at the single cell level within populations of isogenic cells producing different levels and crystalline forms of poly-3-hydroxybutyrate. The exciting technological developments behind O-PTIR have thus allowed us to bypass these sample size limitations.

Despite probing very small levels of biomass, the spectral information provided by O-PTIR for the discrimination of these staphylococci at genus, species and sub-species levels was confirmed to be on par with that provided by FTIR, which was used as a benchmark. For both *S. epidermidis* and *S. capitis*, PC-DFA models were able to unequivocally discriminate between two strains with a test set confirming the robustness of the model. Additionally, the loadings of these models constructed from O-PTIR spectra showed important similarities to the loadings created using FTIR spectra. This allowed for corroboration of the O-PTIR spectral differences found, but also showed that the method was capable of discerning small differences in protein structures within cells, notably through the differences in the amide I peak shape. Although the sample size here is relatively small and does not prove complete strain-level classification ability, these results are a promising demonstration of the ability of O-PTIR to analyse bacteria at the single-cell level.

## Data availability

The data are available on request. Data processing algorithms are available *via*: https://github.com/Biospec/.

## Conflicts of interest

The authors have no conflicts of interest to declare.

## Supplementary Material

AY-016-D4AY00810C-s001
