# Supplementary material for: Benchmarking classification abilities of novel optical photothermal IR spectroscopy at the single-cell level with bulk FTIR measurements
Source: Anal Methods. 2024 Jul 8;16(31):5419–25. doi: 10.1039/d4ay00810c (PMC11308985; doi:10.1039/d4ay00810c)
Supplement: AY-016-D4AY00810C-s001 [file AY-016-D4AY00810C-s001.pdf]

Electronic Supplementary Material (ESI) for Analytical Methods.
This journal is © The Royal Society of Chemistry 2024

Benchmarking Classification Abilities of Novel Optical Photothermal IR Spectroscopy at Single-Cell with Bulk FTIR Measurements

Supplementary Information

Paul I.C. Richardson [a], Malcolm J. Horsburgh [b], Royston Goodacre [a]

[a] Centre for Metabolomics Research, Department of Biochemistry, Cell and Systems Biology, Institute of Systems, Molecular and Integrative Biology, University of Liverpool, BioSciences Building, Crown St, Liverpool, UK

[b] Microbiology Research Group, Institute of Systems, Molecular and Integrative Biology, University of Liverpool, BioSciences Building, Crown St, Liverpool, UK

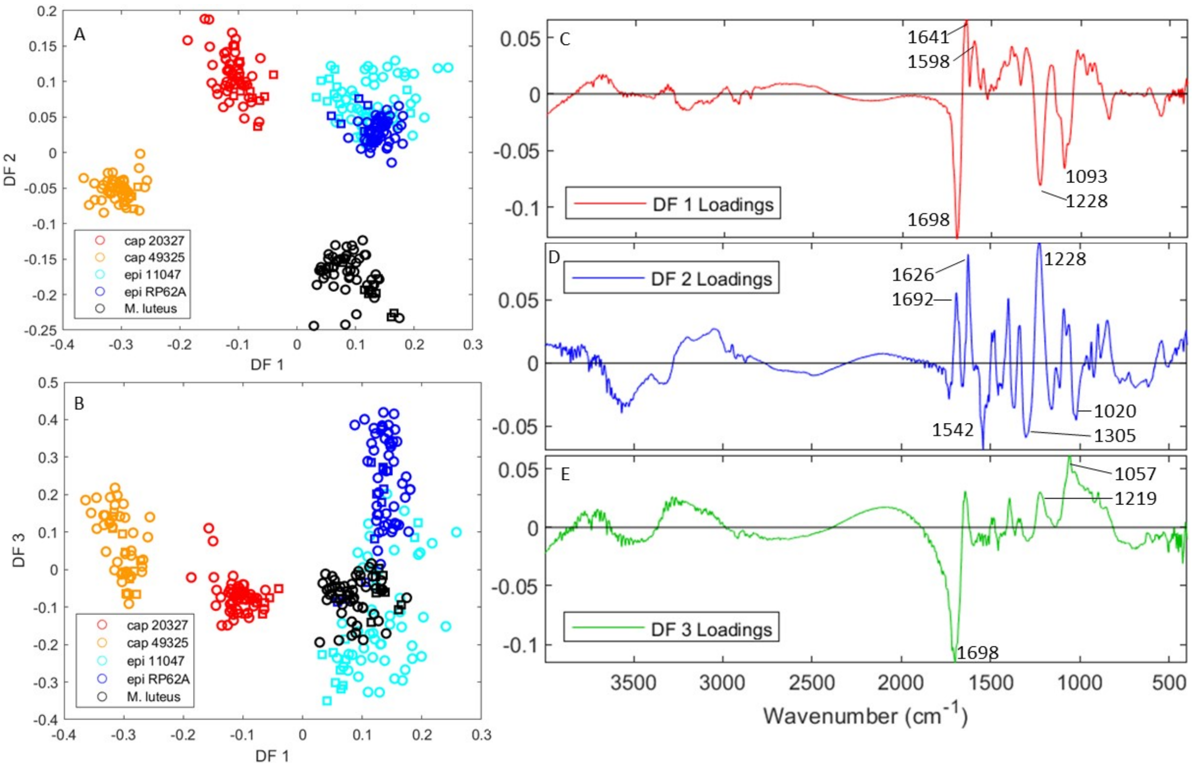

Figure S1: PC-DFA scores and loadings from a model of the full range FTIR spectra for all five tested strains (red: *S. capitis* 20327, orange: *S. capitis* 49325, light blue: *S. epidermidis* 11047, dark blue: *S. epidermidis* RP62A, black: *M. luteus*). The original PCA model contained 7 PCs and these accounted for 97.7% of the total explained variance (TEV). 2A and B represent the scores for DF 1 *vs.* 2 and DF 1 *vs.* 3 respectively, where circles of each colour represent the training set and squares the test set. 2C, D and E represent the loadings for DF 1, 2 and 3 respectively.

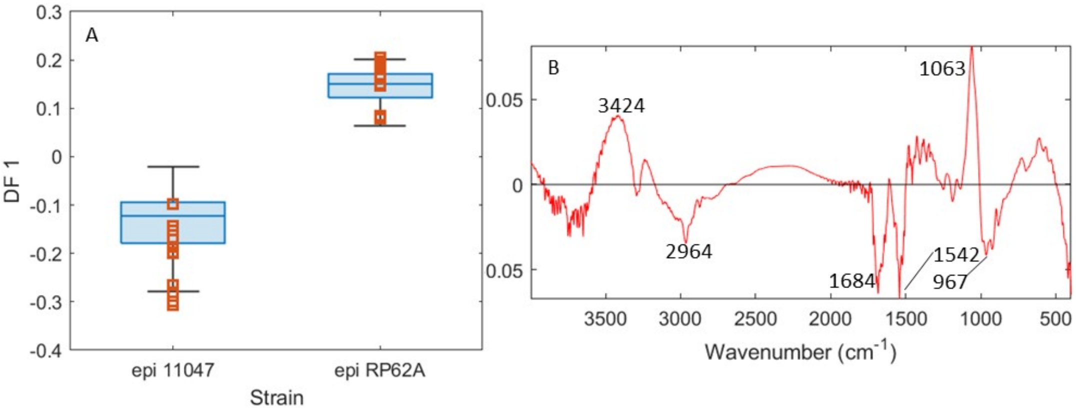

Figure S2: Results of PC-DFA modelling using O-PTIR spectra to compare *S. epidermidis* RP62A and 11047. For scores (S2A), the box and whisker plots depict the interquartile range (edges of the box), the median (middle line in the box) and the extremes of the range not counted as outliers (whiskers) for the training sets for each strain, while orange squares represent the test set. Figure S2B shows the DF 1 loadings. The original PCA contains 6 PCs and explains 97.0% TEV.

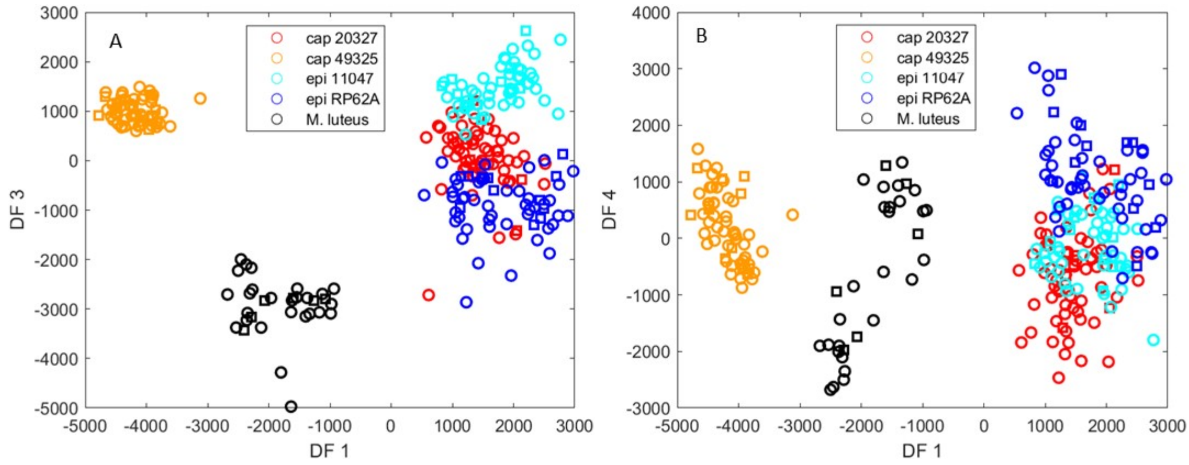

Figure S3: PC-DFA scores and loadings from a model containing O-PTIR spectra for all five tested strains (red: *S. capitis* 20327, orange: *S. capitis* 49325, light blue: *S. epidermidis* 11047, dark blue: *S. epidermidis* RP62A, black: *M. luteus*). Original PCA model contained 7 PCs and explained 96.9% TEV. S3A & B represent the scores for DF 1 *vs.* 3 and DF 1 *vs.* 4, where circles of each colour represent the training set and squares the test set.
